# High-Speed Dicing of SiC Wafers with 0.048 mm Diamond Blades via Rolling-Slitting

**DOI:** 10.3390/ma15228083

**Published:** 2022-11-15

**Authors:** Yuanru Feng, Kenan Li, Zhen Dou, Zhengwen Zhang, Bing Guo

**Affiliations:** 1 State Key Laboratory of Superabrasives, Zhengzhou 450001, China; 2 Zhengzhou Research Institute for Abrasives & Grinding Co., Ltd., Zhengzhou 450001, China; 3 School of Mechatronics Engineering, Harbin Institute of Technology, Harbin 150001, China

**Keywords:** silicon carbide wafer, diamond dicing blades, ultra-thin, rolling-slitting

## Abstract

In this study, an innovative fabrication method called rolling-slitting forming, which forms ultra-thin diamond blades, was presented for the first time. Furthermore, the feasibility of the rolling-slitting forming method when applied to silicon carbide wafer dicing blades was investigated; moreover, the cold-pressing blade samples were manufactured through the conventional process under the same sintering conditions to compare and analyze the manufacturing efficiency, organization and performance. The results show that the new method achieves high-precision and low-thickness dicing blades through continuous production without molds—with the thinnest blades being 0.048 mm thick. Furthermore, the rolling-slitting blade has a unique multiporous heat-conductive matrix structure and in-situ generated amorphous pyrolytic carbon, which can reduce the dicing resistance and contribute to a better cutting quality. In addition, the effects of the dicing parameters on SiC were investigated by using indications of spindle current, dicing chipping size and kerf width during the high dicing process. For a dicing depth of 0.2 mm, the ideal performance of dicing SiC with an ultra-thin blade was achieved at a spindle speed of 22,000 rpm and a feed rate of 5 mm/s. This research provides a new idea for the manufacturing of dicing blades, which can satisfy the demand for ultra-narrow dicing streets of high integration of ICs.

## 1. Introduction

As a novel third-generation semiconductor material, single-crystal silicon carbide is widely used in modern industrial fields, such as new energy vehicles, 5G communications, photovoltaic power generation, rail transit, smart-grids and aerospace, due to its excellent electronic and chemical performances [1,2,3]. SiC showed a high Young’s modulus (about 480 GPa), low coefficient of thermal expansion (3.2–5 × 10^−6^/°C in 25–1400 °C range), high thermal conductivity (30–200 (W/mK)) and super hardness (about 25–30 GPa) [4]. Silicon carbide usually needs to be cut off to fabricate complex microstructures and micro-parts [5]. Most studies in the field of ultra-precision-dicing silicon carbide have focused on electrochemical machining [6,7,8], laser beam machining [9], laser stealth dicing [10,11], water jet cutting and abrasive jet cutting [12,13]. Although the laser stealth dicing and hybrid laser water jet micromachining have high processing efficiencies, thermal ablation defects exist, which impair the processing accuracy. Due to the low economic efficiency and the operational complexity of the above techniques, the diamond dicing blade is an exceptional alternative, but it can also be quite challenging due to the hardness and brittleness of silicon carbide, which inevitably results in chips and surface damage—ultimately leading to yield loss.

Several researchers have attempted to dice silicon carbide [14,15,16,17,18,19,20,21,22,23,24,25], for example, using a diamond wire sawing [14], coating the blade with metallic glass [15,19], applying laser trimming to the blade or adjusting the binder materials [20,24]. Cvetkovic et al. [18] demonstrated the viability of ultra-precision dicing and wire sawing to obtain a high surface quality and minimal edge chipping. Dicing presented an advantage in edge chipping and sidewall roughness. Fujita [15] achieved feasible ductile mode machining of SiC substrates by using a highly rigid PCD blade with a thickness of only 50 μm. The pulsed laser dressed blade has equidistant cutting edges, each with a leading edge and side edges. Chu et al. [19] developed a diamond dicing blade with a metallic glass coating for dicing SiC, and it was shown to significantly reduce the number and size of chips. Yuan et al. [20] performed high-speed micro-cutting of SiC using resin-bonded cutting blades and metal-bonded cutting blades, respectively, indicating that the performance of the metal-bonded cutting blades was inferior to that of the resin-bonded cutting blades. Xie et al. [21] fabricated SiC microchannels with thin diamond grinding wheels and investigated the material removal mechanism of SiC microchannel grinding, and explored the effects of wheel speed, feed speed and grinding depth on SiC geometry and surface quality. Mu et al. [22,23] designed a Ti-rich metal-based blade through a formula that exhibits good cutting quality when cutting silicon carbide; furthermore, they applied a sintered metal-bonded diamond blade for dicing single crystal SiC. It was found that the thickness of the diamond blade has a significant impact on the sintered diamond blade’s dicing ability on SiC—while the minimum thickness of the sintered diamond blade is 200 μm.

The conventional commercial procedures for manufacturing diamond dicing blades include powder cold pressing, hot pressing and sintering, grinding and thinning, and internal and external round machining [24,25]. To improve the utilization of the precious silicon carbide substrate, the width of the dicing street is to be narrowed correspondingly, requiring the thickness of the diamond blade to be increasingly thinner. Nevertheless, there exist significant limitations in the fabrication of high-integrity diamond blades with ultra-thin profiles. On one side, the thickness of the sintered samples prepared by the cold pressing is generally more than 200 μm, to guarantee the uniformity of the material distribution in the direction of the blade thickness [26]. On the other side, warp deformation may easily occur on the blade due to the mechanical removal of the material, which might also cause buckling deformation during the high-speed dicing process and may even lead to blade destruction [27].

More recently, several researchers have revealed the unlimited possibilities of advanced manufacturing techniques in fabricating diamond composites [28,29,30,31,32,33,34,35,36,37,38]. Shaohe Zhang [30] explored the microstructure and property characteristics of Co-based diamond composites prepared by FDMS (fused deposition molding and sintering); however, many interior pores and step effects appeared on the FDMS-prepared samples, and the diamond particles had some thermal damage, which suggests that further optimization of the FDMS process parameters is required. Tao Lin [31] fabricated a vitrified bond diamond grinding wheel by gel casting, which verifiably achieved the performance of a conventional cold-pressed sample, as the gel cast sample had a removal rate of 2.92 g/min and a grinding ratio of 1:152. Jing Lu [32] prepared grinding wheels with a solid structure, triangular structure and lattice structure via DIW (direct ink writing), which indicates that DIW-fabricated grinding wheels featured controlled porosity and improved self-sharpening capabilities. Using SLM technology in AlSi10Mg/diamond composites, Chenchen Tian et al. [33] manufactured a cellular structure that is used for grinding wheels with porosities, which optimizes the porous structure and can improve the performance of the grinding wheel; however, the grinding performance of the samples has not been investigated in detail, leaving the further refinement of the novel design and manufacturing method lacking in application.

Despite the great potential of the advanced manufacturing techniques covered above for forming diamond composites with complex shapes, there are two hindrances to their application in industrial production. First, the products are defect-prone, including low surface roughness, poor precision, diamond graphitization due to high energy sources, and step effects due to stack molding [30]. Second, the equipment used in these techniques is costly and has low production efficiency [19].

For the above-mentioned, a novel fabrication of diamond dicing blades using rolling-slitting and sintering is proposed for the first time. In this study, ultra-thin diamond dicing blades were manufactured by rolling-slitting molding and conventional molding using the same sintering process, respectively. The microstructure and quality of dicing silicon carbide into blades with the two processes were investigated and compared; moreover, the process parameters, such as the spindle speed, feed rate and dicing depth, for cutting silicon carbide were also investigated in this study. To evaluate the cutting performance of the blades, the morphology of the silicon carbide wafer, spindle current and chipping were analyzed, and the appropriate process parameters for dicing silicon carbide with rolling-slitting blades was proposed.

## 2. Materials and Methods

### 2.1. Materials

As shown in Table 1, bronze was selected as a matrix material for the diamond in this study due to its excellent overall performance. To improve the self-sharpening ability of the dicing blade, iron is introduced into the bronze bond, since iron tends to catalyze the graphite formation, and a chemical reaction (formation of carbides, graphite or a solid solution) may occur. The mean grain size of the synthetic diamond abrasive (HuiFeng Diamond Co., Ltd., Zhengzhou, China) used in this study is 10 μm, and the mass fraction is set to 3%.

### 2.2. Fabrication Procedure

**Figure 1:**
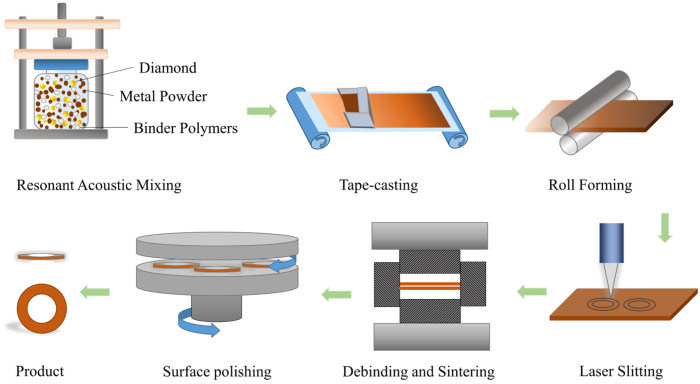
Fabrication procedure of the diamond blade via rolling-slitting.

Figure 1 shows the schematic diagram of the diamond dicing blade that was fabricated by the rolling-slitting and sintering process, which includes resonant acoustic mixing, tape-casting, roll forming, laser slitting, debinding and sintering, and surface polishing.

#### 2.2.1. Mixing Process

**Figure 2:**
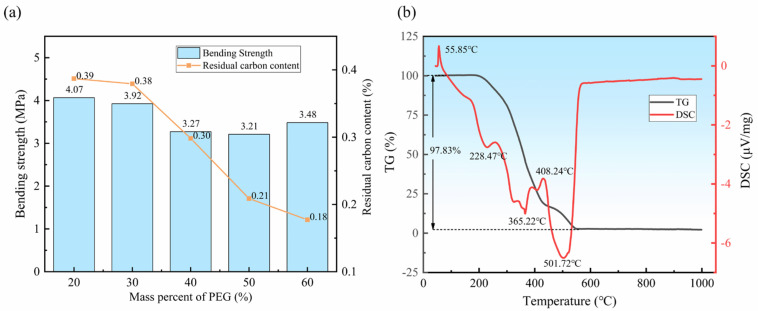
(**a**) The result of bending strength and carbon residue of rolling-slitting green part with different PEG mass fraction; (**b**) TG-DSC curves of binder polymers.

First, the metal powder and diamond abrasives were mixed with binder polymers to form a uniformly mixed slurry through resonant acoustic mixing, due to its low lossless non-paddle mixing and high efficiency. An aqueous clear binder temporary binder system containing polyethylene glycol (PEG) and polyvinyl butyral (PVB) was used to adhere to the metal powder and abrasive diamond. The component ratio of the binder directly affects the rheological performance of the mixed slurry and thus the qualification of the diamond blade. The binder system has to simultaneously fulfill a high bond strength, low carbon residue, low dissolution temperature and good fluidity. Figure 2a shows the relationship between the PEG mass fraction and the performance of the rolling-slitting green part. As the PEG content in the binder increases, the bending strength of the rolling-slitting green part decreases and then increases, while the residue carbon of the green part gradually decreases. When the mass fraction of PEG is 60%, the bending strength of the rolling-slitting green part is 3.48 MPa and the content of residue carbon is only 0.18%, so the overall performance is excellent. To guarantee the binder system has sufficient bond strength and well-releasing properties, before the mixing process, a binder–powder compatibility test and thermal analysis of the binder were conducted by following the procedure that is documented in the literature [38]. The test results show that the wetting angle of the binder-mixed powder is 57.85°, indicating that the binder provides favorable suitability to the mixed powder.

Figure 2b shows the thermo-gravimetry and differential scanning calorimetry (TG-DSC) curves of the binder polymers; moreover, when the temperature exceeds 200 °C, the quality of the binder decreases sharply, and the PEG first undergoes strong thermal decomposition and is then rapidly removed. As the temperature increased, the PVB in the binder was also released and the binder was completely volatilized when the temperature exceeded 500 °C.

#### 2.2.2. Forming and Slitting

In this process, the mixed slurry was poured into the hopper of the tape-casting molding machine, the metal powder and diamond mixing tape were formed on a moving polymer release film, and the thickness of the green tape was controlled by the flat squeegee blade. The dried green tape was transferred into a high-precision rolling compactor to obtain intensive tapes under the pressure of the rollers. In order to obtain high-quality green parts, the casting feed rate and the speed of the roller were set to 400 mm/s and 200 rpm, respectively. Subsequently, we obtained 0.1 mm thick circular green parts with an outer diameter of 53 mm and an inner diameter of 39 mm by laser slitting.

#### 2.2.3. Debinding and Sintering

One-step thermal debinding and hot-press sintering were chosen in this paper to avoid the defects caused by the back-and-forth movement of green parts with the weak powder connection strengths that occur after debinding. First, a small load of 5 kN was applied to the graphite die to ensure that the binder polymer can be decomposed completely. The blade green parts were then heated to approximately 450 °C in 5 min, followed by a maximum temperature of 600 °C for 2 min, while the load was increased to approximately 40 kN. Finally, the temperature was slowly reduced so that the blade would not be fractured by thermal stress. In order to investigate the sintering behaviors and dicing performances of the blade, the corresponding commercial blades that were prepared by cold pressing at 400 kN for 2 min were chosen, and these blades had the same composition and underwent the same sintering process as the rolling-slitting parts.

#### 2.2.4. Surface Polishing

Before surface polishing, the commercially sintered blade sample must be thinned by grinding to remove a large amount of the material, and the thickness of the sample was decreased from about 0.2 mm to about 0.048 mm, while the rolling-slitting sample does not need to be thinned by grinding. The thickness of the sintered rolling-slitting blade sample was around 0.048 mm, which means that it requires no grinding for thinning and material removal, indicating that it only needs to undergo surface polishing to get a qualified sample with high dimensional accuracy and surface roughness.

After the surface-polishing process, internal and external circular machining were also conducted to guarantee that the dicing diamond blade’s installation meets requirements. Finally, the ultra-thin diamond blades with an outer diameter of 52 mm, an inner diameter of 40 mm and a thickness of 0.048 ± 0.005 mm were prepared by the above process.

### 2.3. Analytical Method

The thermal analysis of the binder was performed by an STA449-F3 simultaneous thermal analyzer. The wetting angle of the powder–binder was measured using a video contact angle meter. The fractured cross-section of the manually fractured sintered sample was observed by a scanning electron microscope (SEM, FEI INSPECT S50, FEI, Hillsboro, OR, USA). The elemental composition and distribution were investigated using EDS on the same SEM machine with an electron voltage of 25 kV. The density of the blades was determined by Archimedes’ method. A phase analysis of the surfaces of the green and sintered samples that were prepared by the two formation methods were carried out using an X-ray diffractometer (XRD, BRUKER D8 QUEST ECO, BRUKER, Saarbrücken, Germany).

**Figure 3:**
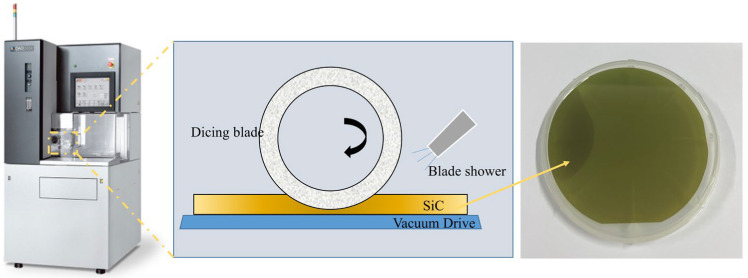
Experimental machine and material.

As shown in Figure 3, the dicing performance of the ultra-thin diamond blade was detected by Disco-DAD-3350 Automatic Dicing Saw. The object of the dicing process was single SiC substrates, and the experiment selected the surface $\left[11\bar{2}0\right]$ of SiC for the dicing experiments.

The detailed dicing test conditions are given in Table 2. An optical microscope was used to observe the SiC surface and measure the width of the dicing street and the size of the edge chippings. To compare and analyze the dicing behavior of the rolling-slitting blade and the cold-pressing forming blade, the spindle current, maximum chip size and kerf width were used as the cutting performance indicators.

## 3. Result and Discussions

### 3.1. Density and Phase Composition of Green and Sintered Samples

**Figure 4:**
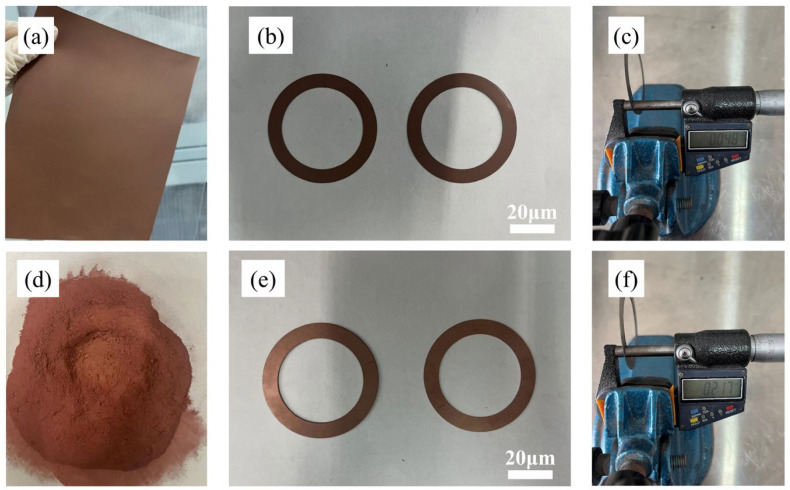
The picture of the precursors, green parts, and sintered parts manufactured by different methods: (**a**–**c**) Rolling-slitting; (**d**–**f**) Cold pressing.

Figure 4 shows pictures of a metal-based diamond ultra-thin blade that was prepared using the rolling-slitting process and the cold-pressing process. As shown in Figure 4c, the thickness of the blade’s sintered part that was produced by the rolling-slitting process can reach 0.048 mm, while the thickness of part from the cold-pressing process is 0.217 mm—making it difficult to further minimize the thickness. It can be seen that the new rolling-slitting process has a number of great advantages when manufacturing extremely thin dicing blades, achieving near-net-shaping of ultra-thin blades, and reducing or even eliminating the need for post-treatment. Meanwhile, the rolling-slitting manufacturing method can obtain the dicing blade shape by laser slitting, which is able to accommodate complex shapes, thereby saving production resources by eliminating the need for steel dies, as well as promising continuously automated and highly efficient generation.

**Figure 5:**
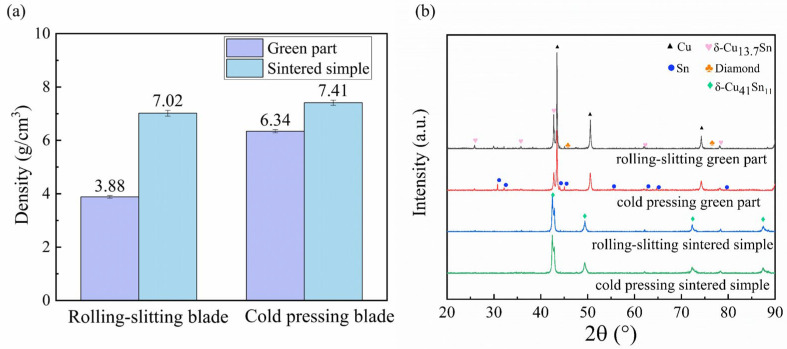
Density and phase composition of green and sintered samples with different forming methods: (**a**) Density; (**b**) Phase composition.

The density and phase composition of the green and sintered samples under the two different forming methods is shown in Figure 5. It can be seen that the density of the rolling-slitting green part is only 3.88 g/cm^3^, which differs significantly from the density of the cold-pressed one, which is 6.34 g/cm^3^; moreover, the density of the rolling-slitting sintered sample is 7.02 g/cm^3^, which is moderate compared with the density of the cold-pressed sintered sample, which is 7.41 g/cm^3^. This slight density variation was attributable to the removal of the binder during the debinding and sintering process. Early in the debinding and sintering process, the organics gradually thermally decompose, leaving pores. As the temperature and pressure continuously increase, the metallic bond gradually forms a sintering neck and undergoes diffusion, forming a metallurgical bond. The metallic bond shrinks accordingly during cooling in the later sintering, gradually wrapping the diamond and experiencing a significantly higher densification.

As the composition, organization and phase structure of the material in the blade play an important role in the service performance of the blade, we used the phase composition of the green and sintered samples of the dicing blades that were prepared by the different forming methods in order to compare the differences between the two blades. Figure 5b shows the phase composition of the green and sintered samples of the dicing blades that were prepared by the different forming methods. Strong diffraction peaks corresponding to copper, tin, copper–tin solid solution and diamond were clearly identified for all the green parts, whereas clear strong diffraction peaks for copper–tin solid solution and diamond were identified for the sintered samples. The phase composition and content of the sintered samples prepared by the two forming methods were nearly identical, with no obvious graphite peaks detected in the rolling-slitting sintered samples, which corresponds to the low residual carbon of the binder at the current sintering temperature mentioned in Section 2.

### 3.2. Microstructure of the Dicing Blade with Different Forming Methods

As the three components of the blade, the binder, the pore and the diamond directly affect the dicing performance of the blade. The microstructure of the blade is usually characterized by the adhesion behavior and porosity of the binder with the diamond, which can direct the appropriate design of the blade.

**Figure 6:**
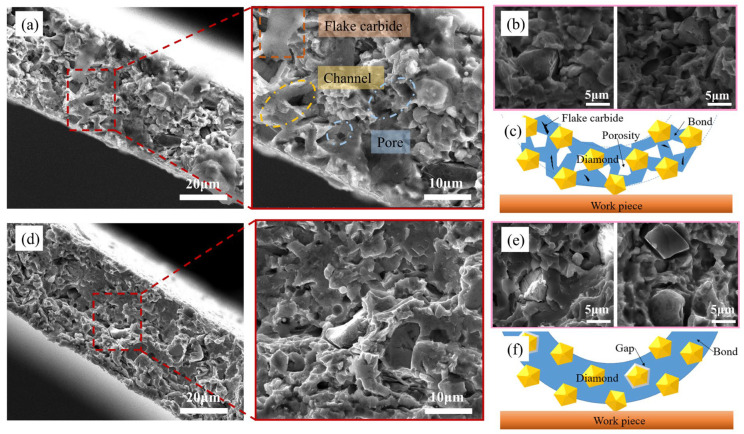
Microstructure of the dicing blades with different forming methods; (**a**–**c**) Rolling-slitting; (**d**–**f**) Cold pressing.

Figure 6 shows that the scanning fracture microstructures of the blade samples under the two forming methods differ distinctly. The fracture of the cold-pressed samples was characterized by a quasi-cleavage fracture with only a few dimples, which had a relatively flat fracture pattern that featured high structural densification and low porosity. As for the rolling-slitting blade, it shows a cleavage fracture, which is consistent with the brittle fracture, and the inner structure of the samples was relatively fragmentary with an inferior degree of densification. Further study of the sintered sample’s fracture from rolling-slitting forming revealed that irregular thin layers of carbide, microchannels and tiny pores were diffusely distributed diffusely within, which is likely attributable to the addition of binder during the forming process. A multitude of pores that were generated by the binder emerged from the green parts during the post-debinding and -sintering processes. A very small portion of the binder, which was insufficiently decomposed, remained in the sample to form flake carbides.

Figure 6b shows the diamond microstructure of the insert prepared by the rolling-slitting molding process, and Figure 6e shows the diamond microstructure of the insert prepared by the cold-pressing molding process. It can be seen that the diamond and the bond matrix in the cold-pressed blades are at the extremes, i.e., one is the diamond–bond solid mechanical engagement in the dense area of the bond and the other is a minor segment of the diamond surrounded by a significantly wider gap. This causes the diamond particles to flake off during the cutting process, thereby resulting in a dull blade with difficulties in achieving self-sharpening, or are simply dislodged, resulting in large particle scratches. Blades prepared by the rolling-slitting process have a more homogeneous organization, with tiny pores diffusely distributed between the diamond and the bond, whereby the diamond is bonded and stabilized by the metal bond containing the pores; moreover, these tiny pores also provide capacity for the filling of coolant and the removal of chips. As a result, these rolling-slitting blades may offer significant advantages for the cutting of hard and brittle materials compared to the cold-pressed blades.

Figure 6c,f gives schematic illustrations of blade cutting through the different formation methods. In summary, the rolling-slitting sample has distinctive internal characteristics and structures compared with the cold-pressed sample. On the one hand, the metal bond species of the rolling-slitting sample has diffusely distributed fine pores, which can facilitate cooling and chip removal, promote diamond exposure, and improve the self-sharpening of the blade. On the other hand, the thermal decomposition of the binder leaves some of the carbide flaky, which generates a lubricating layer on the working surface so as to decrease the dicing resistance.

### 3.3. Influence of Dicing Parameters on SiC Surface Quality

For ultra-high-speed precision dicing of hard and brittle materials, chip fracturing provides an important failure mechanism and material removal method [39]. The wafer dicing process, which includes the spindle speed, feed rate and dicing depth, has an important influence on die quality. The dicing diamond blade has to be coordinated with the appropriate dicing process in order to fulfil the blade’s performance [40,41,42,43]. In order to compare the dicing performance of the blades that were prepared by the two formation methods under different conditions, the influence of the spindle speed, feed rate and cutting depth on the dicing quality was studied and the optimal process parameters for cutting silicon carbide with the diamond blade were obtained in this chapter.

#### 3.3.1. The Effects of the Spindle Speed

**Figure 7:**
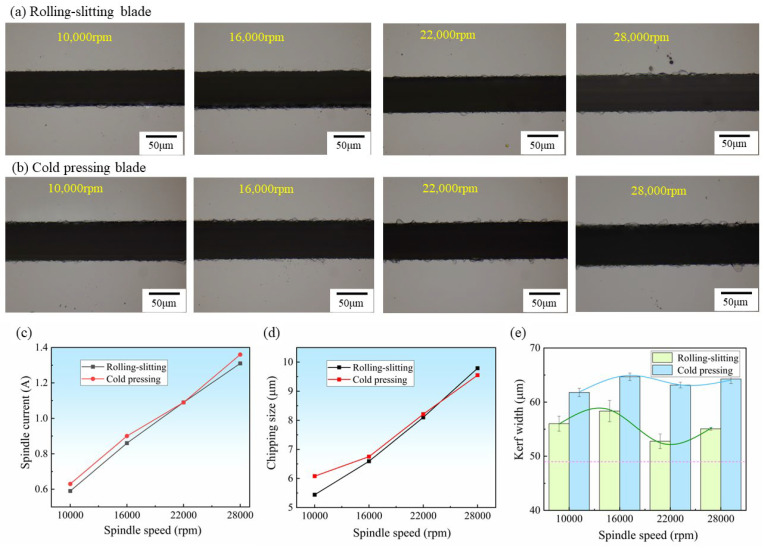
Result of SiC wafer diced at different spindle speeds, feed rate *vf* = 1 mm/s dicing depth *ap* = 0.2 mm: (**a**,**b**) Surface morphology of SiC, (**c**) Spindle current, (**d**) Maximum chipping size, (**e**) Kerf width.

Like any other hard and brittle material, the dicing quality of silicon carbide material can be very sensitive to the spindle speed [34]. Figure 7a,b shows the surface morphology of silicon carbide substrate dicing at different spindle speeds using the rolling-slitting blade and cold-pressed blade, respectively. It can be seen that the kerf width and chipping size of the rolling-slitting blade are preferable to the cold-pressed blade under the same conditions, and the cold-pressed blade incurs continuous chipping during the high-speed cutting process. Figure 7c–e shows the spindle current, chipping size and kerf width during the dicing process at different spindle speeds. As the spindle speed increases, the dicing current rises linearly, while the spindle current of the rolling-slitting blade is slightly smaller than that of the cold-pressed one. The spindle current during the dicing is a symbol of the cutting force, the lower spindle current indicates the smaller cutting force during the cutting and the better sharpness of the blade [42]. As the spindle speed increases, the load on the blade grows and the cutting force gradually increases. Since the thickness of the blade is only 48 μm, the damage to the cutting process is slight and the change in cutting resistance is not apparent. When the spindle speed increases, the chipping size of the blade cutting tends to increase. The maximum chipping size of the blades prepared by the two processes is similar. This may be due to the finer diamond particle size selected for the blades; the smaller it is, the smaller the volume of the scribing workpiece—and the size of the chipping is not sensitive to changes [17].

However, the effect of spindle speed on the kerf width is significantly different from the results of the other two items, following a tendency to increase, then decrease, and thereafter increase. As the spindle speed increased from 10,000 rpm to 16,000 rpm, the kerf width of the rolling-slitting blade increased from 56.02 μm to 58.34 μm, and the kerf width of the cold-pressed blade increased from 61.78 μm to 64.68 μm; this could be attributed to the fact that as the rotational speed increased, the blade introduced more crosswise cracking toward the perimeter and increased the removal of silicon carbide. Interestingly, as the spindle speed further increased to 22,000 rpm, the kerf width gradually decreased. This could be because, in the case of an even higher spindle speed, the dicing distance of single abrasive grains decreased sharply, and the vertical grinding force tended to be smaller. Xie et al. [21] observed a similar phenomenon when grinding silicon carbide microchannels, and they concluded that the grinding distance of single abrasive grains decreased at higher speeds, which reduced the vertical cutting force of a single abrasive, resulting in less material removal and a narrower kerf width. Nevertheless, when the spindle speed increased up to 28,000 rpm, the kerf width increased again, which is probably because the blade is vulnerable to instability during cutting with a high spindle speed; in short, the dicing blade body vibrates away from the blade-moving forward axis so that the width of the cutting slit broadened against the increase in amplitude.

Additionally, the kerf width of the rolling-slitting blade is relatively narrow compared to the cold-pressed one under an identical condition. The reason for the above phenomenon is that the unique three-dimensional porous heat-conducting structure of the rolling-slitting blade achieves a significant advantage in cutting hard and brittle materials; whereas its diamond is bridged by a space-continuous porous metal matrix that is easily detached. Furthermore, the diamond of the cold-pressed blade is tightly covered by a dense metal matrix that cannot be detached easily, and the blade swings back and forth during the cutting process due to insufficient cutting capacity after times of cutting, flattening and dulling, resulting in a wider kerf. Similarly, Adachi et al. [43] found comparable phenomena when they used the casting method and pulsed electric current sintering (PECS) method to manufacture cutting blades.

#### 3.3.2. The Effects of the Dicing Depth

**Figure 8:**
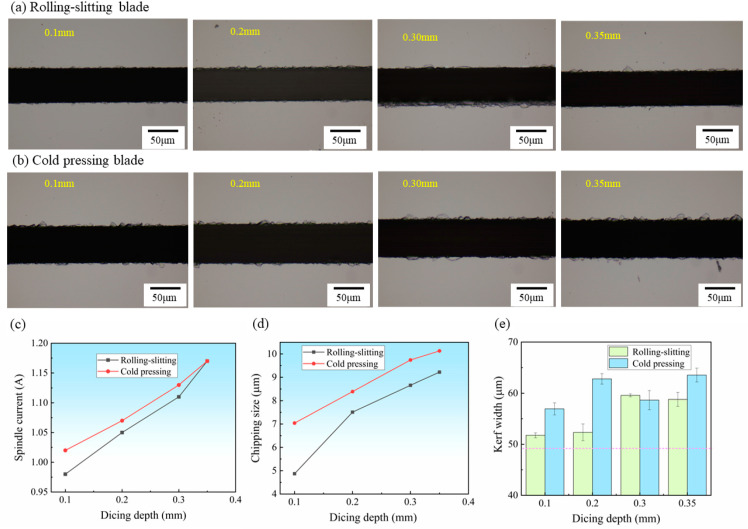
Result of SiC wafers diced at different dicing depth, Spindle speed *n* = 22,000 rpm, feed rate *vf* = 1 mm/s: (**a**,**b**) Surface morphology of SiC, (**c**) Spindle current, (**d**) Maximum chipping size, (**e**) Kerf width.

Dicing depth has a significant impact on the quality of material dicing. As the dicing depth increases, the area of the material being removed increases and the dicing resistance increases. Figure 8a,b shows the surface morphology of silicon carbide substrate cutting at different dicing depths using a cold-pressed blade and rolling-slitting blade, respectively. It can be seen from the figure that the effect of the dicing depth on the cutting quality is less than the other two items, and the quality of the rolling-slitting blade for cutting seams is better than that of the cold-pressed blade. As Figure 8c shows, the dicing depth has a greater influence on the spindle current. When the dicing depth increased from 0.10 mm to 0.35 mm, the spindle current increased sharply with a significant growth rate, and the chipping size and kerf depth increased slightly with a gentle growth rate. Due to the heightening of the cutting depth, the cutting depth of a single particle of diamond increases, the volume of the scribing workpiece increases, the load of the blade increases sharply, and the spindle current also rapidly increases. The blade deformation and chipping occur easily due to stress, and the chipping size and kerf width tend to increase slightly due to this.

#### 3.3.3. The Effects of the Feed Rate

**Figure 9:**
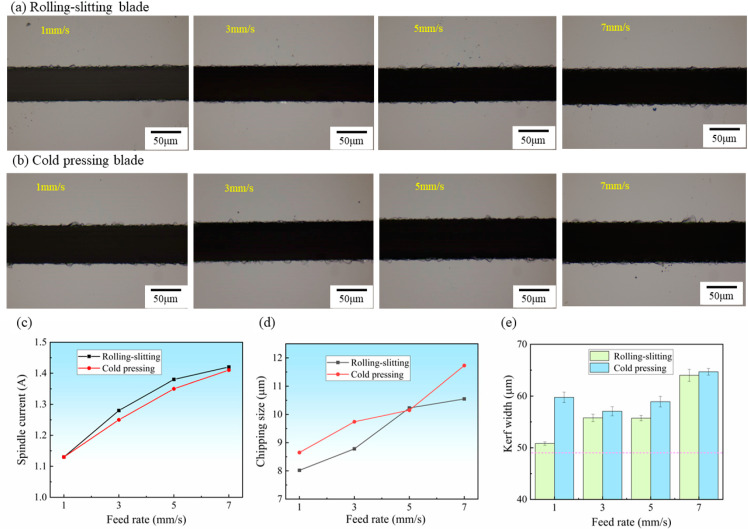
Result of SiC wafers diced at different feed rates, Spindle speed *n* = 22,000 rpm dicing depth *ap* = 0.2 mm: (**a**,**b**) Surface morphology of SiC, (**c**) Spindle current, (**d**) Maximum chipping size, (**e**) Kerf width.

Figure 9a,b shows the surface morphology of silicon carbide substrate cutting at different feed rates using a cold-pressed blade and rolling-slitting blade, respectively. As can be seen, the number, size and surface area of chips tended to increase with the increase in the feed rate, especially when the feed rate increased to 7 mm/s, wherein the cutting chipping and kerf width of both blades increased dramatically, and the cold-pressed blade presented relatively large chips that were over 10 mm in size; this can likely be explained by the poor self-sharpening ability of the cold-pressed blade. As it was hard to automatically refresh a few diamond particles due to the powerful binding agent coating, which protruded from the surface of the blade during dicing, the blade became blunt or even overloaded, resulting in a noticeable growth of dicing chipping.

Figure 9c–e shows the effect of the feed rate on the spindle current, chip size and kerf width. With an increase in the feed rate, the spindle current, chip size and kerf width tend to increase. At a feed rate of 7 mm/s, both blades cut silicon carbide with a kerf width of 60 μm or more—with a relative kerf width of 1.20. The blades were overloaded along with the continuous increase in the cutting current, and the workpiece experienced higher stresses, leaving it susceptible to chipping. With the increase in the dicing feed rate, the single diamond scribing range increased, and the diamond failed to fall off in a timely manner, given that it was prone to heat concentration, which increased the load of the blade and eventually resulted in the easy production of larger chipping, cracking and other defects [25].

The feed rate directly affects the dicing width and the chipping size as well as the dicing efficiency. For production applications, silicon carbide wafers are required to have chipping of fewer than 15 μm and a dicing width of fewer than 60 μm, and it is crucial that the feed rate be fast in order to obtain chips productively. In summary, for a dicing depth of 0.2 mm, the ideal SiC dicing with an ultra-thin blade was achieved at a spindle speed of 22,000 rpm and a feed rate of 5 mm/s.

In comparison with the traditional commercial cold-pressing-formed dicing blades, the porous rolling-slitting dicing blades possess preferential advantages in terms of dicing speed, stability and heat dissipation capability. Overall, the rolling-slitting forming technology offers a novel way to obtain homogeneous, well-structured diamond blades, which have excellent potential for dicing hard and brittle materials.

## 4. Conclusions

In this research, a metal-based ultra-thin dicing blade was prepared using the rolling- slitting forming technique and was compared with a blade obtained by cold pressing to investigate their microstructural characteristics and properties, as well as the SiC dicing quality regulation. The main conclusions that were drawn are as follows:

(1) An innovative fabrication method named rolling-slitting for ultra-thin diamond dicing blades was proposed for the first time. The new fabrication method can achieve high precision and thin thickness, reaching up to 0.048 mm without grinding-thinning. This method facilitates cost-effective and continuous production without dies.
(2) The porous structures formed in situ of rolling-slitting diamond blades are realized, and residual pyrolysis carbon forms a lubrication film that can reduce dicing resistance and achieve ultra-narrow and self-sharpening during the dicing of silicon carbide chips.
(3) The dicing performance of rolling-slitting blades and cold-pressed blades was comparatively studied and the influence of the dicing parameters on the quality of silicon carbide cutting was investigated as well. For a dicing depth of 0.2 mm, the ideal SiC dicing with an ultra-thin blade was achieved at a spindle speed of 22,000 rpm and a feed rate of 5 mm/s.

## 5. Patents

A green precision continuous forming system and process for ultra-thin metal grinding wheels [P]. Henan Province: CN111618750A, 2020-09-04.

## Figures and Tables

**Table 1:** The formula for Ultra-Thin Diamond Dicing Blade.

Ingredient	Cu	Sn	CuSn10	Fe	Diamond
Particle size (μm)	8	15	10	10	8–12
Percent wt.%	65	15	15	2	3

**Table 2:** Dicing test conditions.

Apparatus	Automatic Dicing Saw	DISCO-DAD-3350
Diamond blade	Size	52 × 0.048 × 40
Single silicon carbide	Type	4H-SiC-350 µm
	Dicing direction	$11\bar{2}0$
Dicing parameters	Spindle speed	10,000, 16,000, 22,000, 28,000 rpm
	feed rate	1, 3, 5, 7 mm/s
	Dicing depth	0.1, 0.2, 0.3, 0.35 mm

## Data Availability

Not applicable.

## References

[1] Lee H., Smet V., Tummala R. (2019). A review of SiC power module packaging technologies: Challenges, advances, and emerging issues. IEEE J. Emerg. Sel. Top. Power Electron..

[2] Wu J., Ren N., Guo Q., Sheng K. (2020). A comparative study of silicon carbide merged PiN schottky diodes with electrical-thermal coupled considerations. Materials.

[3] Nakamura D., Gunjishima I., Yamaguchi S., Ito T., Okamoto A., Kondo H., Onda S., Takatori K.J.N. (2004). Ultrahigh-quality silicon carbide single crystals. Nature.

[4] Rakshit R., Das A.K. (2019). A review on cutting of industrial ceramic materials. Precis. Eng..

[5] Goel S. (2014). The current understanding on the diamond machining of silicon carbide. J. Phys. D Appl. Phys..

[6] Han W., Kunieda M. (2020). Precision electrochemical machining of tungsten micro-rods using wire electrochemical turning method. Int. J. Adv. Manuf. Technol..

[7] Nishikawa H., Okada Y., Yamamura K., Matsuyama S., Yamauchi K. (2014). Dicing of SiC wafer by atmospheric-pressure plasma etching process with slit mask for plasma confinement. Materials Science Forum.

[8] Zhang Y., Li R., Zhang Y., Liu D., Deng H. (2019). Indiscriminate revelation of dislocations in single crystal SiC by inductively coupled plasma etching. J. Eur. Ceram. Soc..

[9] Zhang Z., Wen Z., Shi H., Song Q., Xu Z., Li M., Hou Y., Zhang Z. (2021). Dual Laser Beam Asynchronous Dicing of 4H-SiC Wafer. Micromachines.

[10] Wang L., Zhang C., Liu F., Zheng H., Cheng G. (2022). Ultrafast pulsed laser stealth dicing of 4H-SiC wafer: Structure evolution and defect generation. J. Manuf. Process..

[11] Yang B., Wang H., Peng S., Cao Q. (2022). Precision Layered Stealth Dicing of SiC Wafers by Ultrafast Lasers. Micromachines.

[12] Feng S., Huang C., Wang J., Zhu H. (2018). Material removal of single crystal 4H-SiC wafers in hybrid laser-waterjet micromachining process. Mater. Sci. Semicond. Process..

[13] Miao X., Qiang Z., Wu M., Song L., Ye F. (2018). The optimal cutting times of multipass abrasive water jet cutting. Int. J. Adv. Manuf. Technol..

[14] Hardin C.W., Qu J., Shih A. (2004). Fixed abrasive diamond wire saw slicing of single-crystal silicon carbide wafers. Mater. Manuf. Process..

[15] Fujita T., Izumi Y., Watanabe J. (2019). Ultrafine ductile-mode dicing technology for SiC substrate with metal film using PCD blade. J. Adv. Mech. Des. Syst. Manuf..

[16] Gao A., Huang W., Han R. (2020). Research on SiC wafer dicing technology. Equpiment Electron. Prod. Manuf..

[17] Ya C., Con Z., Mei Z., Guo Y. (2017). Experimental study of surface quality of monocrystalline SiC cut by ultra-thin diamond saw blade. Superhard Mater. Eng..

[18] Cvetković S., Morsbach C., Rissing L. (2011). Ultra-precision dicing and wire sawing of silicon carbide (SiC). Microelectron. Eng..

[19] Chu J.P., Lai B.Z., Yiu P., Shen Y.L., Chang C.W. (2020). Metallic glass coating for improving diamond dicing performance. Sci. Rep..

[20] Wang X., Yuan Z., Zhuang P., Wu T., Feng S. (2021). Study on precision dicing process of SiC wafer with diamond dicing blades. Nanotechnol. Precis. Eng..

[21] Xie Y., Deng D., Pi G., Huang X., Zhao C. (2020). Fabrication of silicon carbide microchannels by thin diamond wheel grinding. Int. J. Adv. Manuf. Technol..

[22] Wang P., Li M., Gao L., Meng H., Mu D. The evaluation of chipping on single-crystal silicon carbide (SiC) dicing machining using sintered diamond blades. Proceedings of the 2nd International Conference on Mechanical, Electronics, and Electrical and Automation Control (METMS 2022).

[23] Li M., Mu D., Huang S., Wu Y., Meng H., Xu X., Huang H. (2022). Ultrathin diamond blades for dicing single crystal SiC developed using a novel bonding method. J. Manuf. Process..

[24] Yuan Z., Feng S., Wu T. (2022). Preparation and characterization of ultra-thin dicing blades with different bonding properties. Int. J. Adv. Manuf. Technol..

[25] Yuan Z., Cheng K., Zhang Y., Hu J., Zheng P. (2019). Investigation on the fabrication of dicing blades with different sintering methods for machining hard-brittle material wafers. Proc. Inst. Mech. Eng. Part B J. Eng. Manuf..

[26] Jie Y., Shuyi G. (2016). Design of ultrathin diamond tool powder feeding system. Diam. Abras. Eng..

[27] Zou Q., Zhang C., Li Y., Li K. (2022). Research present situation of machining deformation of ultra-thin dicing blades. Diam. Abras. Eng..

[28] Denkena B., Krödel A., Harmes J., Kempf F., Griemsmann T., Hoff C., Hermsdorf J., Kaierle S. (2020). Additive manufacturing of metal-bonded grinding tools. Int. J. Adv. Manuf. Technol..

[29] Kasonde M., Kanyanta V. (2016). Future of superhard material design, processing and manufacturing. Microstructure-Property Correlations for Hard, Superhard, and Ultrahard Materials.

[30] Su Z., Zhang S., Liu L., Wu J. (2021). Microstructure and performance characterization of Co-based diamond composites fabricated via fused deposition molding and sintering. J. Alloys Compd..

[31] Lin T., Liu S., Ji Z., Shao H., Hao J. (2020). Vitrified bond diamond grinding wheel prepared by gel-casting with 3D printing molds. Diam. Relat. Mater..

[32] Huang J., Lu J., Wang Y., Ma Z. (2021). Fabrication of porous structure vitrified bond diamond grinding wheel via direct ink writing. Ceram. Int..

[33] Tian C., Li X., Li H., Guo G., Wang L., Rong Y. (2019). The effect of porosity on the mechanical property of metal-bonded diamond grinding wheel fabricated by selective laser melting (SLM). Mater. Sci. Eng. A.

[34] Wang C., Wang D., Tian C., Wang L., Rong Y., Li X. (2021). Grinding performance evaluation of 3D-printed porous metal-bonded grinding wheel in BK7 glass grinding. Int. J. Adv. Manuf. Technol..

[35] Tian C., Li X., Chen Z., Guo G., Wang L., Rong Y. (2020). Study on formability, mechanical property and finite element modeling of 3D-printed composite for metal-bonded diamond grinding wheel application. J. Manuf. Process..

[36] Li M., Huang J., Fang A., Mansoor B., Pei Z., Ma C. (2021). Binder jetting additive manufacturing of copper/diamond composites: An experimental study. J. Manuf. Process..

[37] Gan J., Gao H., Wen S., Zhou Y., Tan S., Duan L. (2020). Simulation, forming process and mechanical property of Cu-Sn-Ti/diamond composites fabricated by selective laser melting. Int. J. Refract. Met. Hard Mater..

[38] Li M., Du W., Elwany A., Pei Z., Ma C. (2020). Metal binder jetting additive manufacturing: A literature review. J. Manuf. Sci. Eng..

[39] Lin J., Cheng M. (2014). Investigation of chipping and wear of silicon wafer dicing. J. Manuf. Process..

[40] Xue M., Chen T., Zhang X., Gao L., Li M. Effect of blade dicing parameters on Die strength. Proceedings of the 2018 19th International Conference on Electronic Packaging Technology (ICEPT).

[41] Gamkrelidze S., Trofimov A., Shchavruk N.J.R.M. (2017). Effect of diamond dicing of SiC device wafers on the technical and operational parameters of monolithic integrated circuits. J. Russ. Microelectron..

[42] Yuan Z., Hu J., Wen Q., Cheng K., Zheng P. (2018). Investigation on an innovative method for high-speed low-damage micro-cutting of CFRP composites with diamond dicing blades. Materials.

[43] Adachi T., Matsumaru K., Ishizaki K. (2006). Fabrication of highly efficient dicing blade for cutting Al2O3-TiC composite. J. Ceram. Soc. Jpn..

